# Anserine, HClO-scavenger, protected against cognitive decline in individuals with mild cognitive impairment

**DOI:** 10.18632/aging.202535

**Published:** 2021-01-20

**Authors:** Nobutaka Masuoka, Chenxu Lei, Haowei Li, Noriko Inamura, Shigenobu Shiotani, Nobuya Yanai, Kenichiro Sato, Keisuke Sakurai, Tatsuhiro Hisatsune

**Affiliations:** 1 Graduate School of Frontier Sciences, The University of Tokyo, Kashiwa, Japan; 2 Community Hearth Promotion Laboratory, Mitsui Fudosan, Co., Ltd., Kashiwa, Japan; 3 Urban Design Center Kashiwanoha (UDCK), Kashiwa, Japan; 4 Research Division, Tokai Bussan Co., Ltd., Tokyo, Japan

**Keywords:** Alzheimer’s disease, mild cognitive impairment, myeloperoxidase, hypochlorous acid, anserine

## Abstract

Neuroinflammation has been recognized as a promising target when considering strategies for treating AD. In particular, it has been shown that neutrophils and MPO-mediated neuroinflammatory responses with the production of HClO play a role in the progression of AD. In this study, we aimed to evaluate the effects of anserine, a scavenger of HClO, on the protection of cognitive declines in persons with MCI. Fifty-eight elderly volunteers were screened, and 36 MCI individuals were assigned either to an active arm, who received 500 mg anserine per day, or a placebo arm, for 12-weeks. To assess cognitive function, we performed MMSE at baseline and after the ingestion. The data of the MMSE for 30 subjects who completed the follow-up tests were analyzed. A significant difference was detected in the change score of MMSE between the active arm (1.9 ± 2.0; n = 15) and the placebo arm (0 ± 2.8; n = 15) (*p* = 0.036). After the correction with the daily intake of anserine, the significance was elevated (*p* = 0.0176). Our results suggest that anserine protects elderly persons with MCI from cognitive declines by suppressing MPO-mediated neuroinflammatory responses.

## INTRODUCTION

By 2050, more than 130 million people are estimated to have dementia worldwide [1]. It is thought that there will be nearly 10 million new cases every year [2]. AD, the most frequent cause of dementia, is a neurodegenerative disease. In AD, the accumulation of cerebral amyloid-beta has usually been occurring for more than 20 years before the onset of dementia [3]. At the dementia stage, neuronal damage progresses together with the accumulation of tau protein [3]. The necessity of targeting preclinical or prodromal AD in clinical trials for preventing dementia due to AD is now widely recognized [4, 5]. However, no clear evidence of pharmacological treatments or daily life improvement for preventing the development of dementia has been demonstrated [6, 7].

In recent years, the role of neuroinflammation in the pathogenesis of AD has received attention [8–10]. In addition to cells in brain tissue, blood vessels and blood cells also have potent effects on neuroinflammation. The pursuit of drug targets other than the inhibition of senile plaques accumulation or neurodegeneration would be hoped [11]. The innate immune system contributes to neuroinflammation [9, 12, 13]. It has been shown that MPO-mediated inflammatory responses caused by the production of HClO radicals play a role in the progression of AD [14–18]. In AD patients, it has been confirmed that MPO expression increases in brain tissue [14], and it has also been reported that the MPO level increases in the blood [18]. In addition, in an Asian cohort study, MPO rs2333227 polymorphism was positively associated with AD risk and MPO accumulation in the plasma [15]. Animal model studies have also provided evidence that MPO is involved in the development of AD. It has also been reported that MPO activity inhibition improves cognitive functions in the AD model mice [19, 20].

Anserine is among the molecular species called histidine-containing dipeptides [21–25]. They exist at high concentrations in the muscle of some vertebrate species and are known to have antioxidative activity toward HClO radicals [21, 26–28]. The beneficial effects of ACS on cognitive functions were demonstrated in previous trials, in which the test material derived from the chicken extract was used to provide the study subjects 1000 mg mixture of anserine and carnosine (approx. 3: 1 weight ratio) per day, for 3 - 12 months [29–34]. It was shown that ACS suppressed cognitive decline in normal subjects [29–31, 33, 34] and might promote reversion to cognitive-normal in MCI subjects [32]. Carnosinase, present in serum and brain, is thought to degrade carnosine quickly when orally taken [21, 35]. Therefore, we hypothesized that anserine would function as an active compound. In an animal model, anserine alone protects against cognitive decline in AD model mice [36]. In the present study, we conducted a randomized, double-blind, placebo-controlled trial in which the test substance was anserine, and the subjects were with MCI.

## RESULTS

### Study individuals

**Figure 1:**
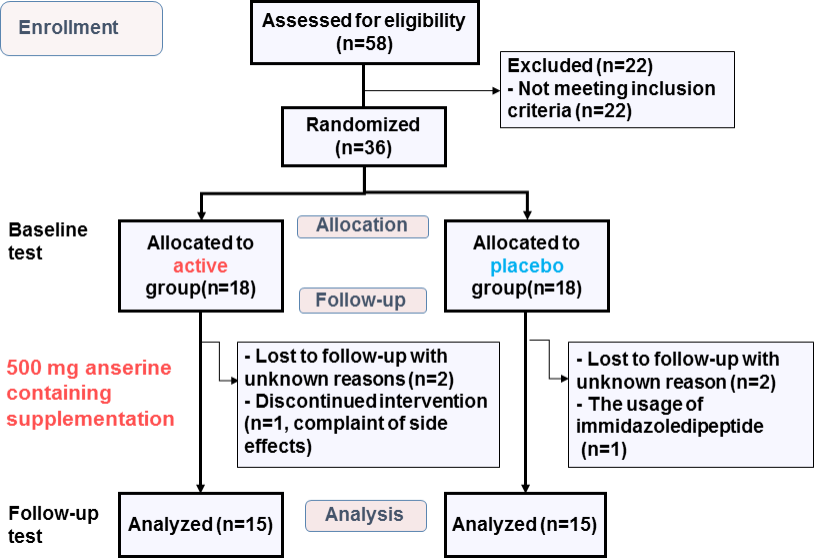
**Flow diagram showing the number of subjects during the study.** Baseline test: The test at the start of the intervention. Follow-up test: The test at twelve weeks after the start.

Of 58 participants, the conditions of 36 individuals were diagnosed as MCI, considering their history and presence at an interview with the assistance of a psychological test battery. Both the active arm and placebo arm registered 18 after randomization, as shown in Figure 1. Among them, 30 individuals completed the examinations at follow-up (15 in the active arm and 15 in the placebo arm). Table 1 shows the characteristics. All reported an ingestion rate of over 90% on their self adherence records. The amounts of anserine and carnosine intake from daily meals estimated from a dietary survey were found to be equivalents between the two arms.

**Table 1:** Characteristics of the study subjects who completed the study at baseline.

	**Active group**	**Placebo group**	**p value**
Age	74.5±4.6 ^a^	72.0±5.2	0.17
Gender (M/F)	11/4	8/7	0.26
BMI	21.9±2.2	21.4±2.1	0.49
Years of education	15.4±2.5	15.0±1.9	0.62
Daily anserine (mg)	335±55	368±42	0.71
Daily carnosine (mg)	162±33	171±20	0.86

^a^Average ± Standard Deviation.

### Test formulae

**Figure 2:**
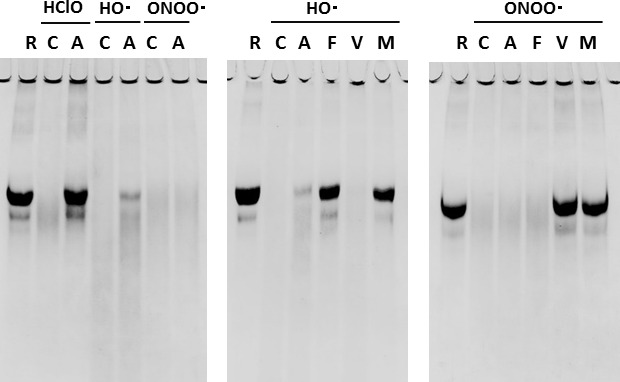
**Anserine as a scavenger for hypochlorous acid, HClO.** HClO (5.0mM), HO Â· (10mM), ONOO Â· (5.0mM). R: Reference protein, C: Control, A: Anserine, F: FA, V: VC, M: Mixture.

The test formula for the active arm contains purified anserine (> 93%) from salmon meat. The active arm's supplement consisted of anserine powder, dextrin, maltose, sweeteners (stevia and sucralose), flavor, vitamin C, citric acid, and ferulic acid. Table 2 shows the amounts contained in a package. The anserine in the active food was replaced by dextrin in the food in the placebo arm. The supplement provided for both arms was indistinguishable by sight, smell, or taste. In an antioxidant assay, anserine was shown to be a potent HClO scavenger. It specifically removed the toxicity of HClO (Figure 2). Ferulic acid scavenged the toxicity of OH radicals, and vitamin C did that of ONOO radicals. We provided two packages a day so that the active arm subjects would ingest 500 mg of anserine per day, 250 mg both in the morning and the evening.

**Table 2:** Test Formulae.

**Ingredient**	**Active**	**Placebo**
Anserine	250 mg	0 mg
Ferulic Acid (FA)	15 mg	15 mg
Vitamin C (VC)	75 mg	75 mg
Citric Acid	200 mg	200 mg
Maltose	500 mg	500 mg
Sweeteners	11 mg	11 mg
Flavor	7 mg	7 mg
Dextrin	942 mg	1192 mg
Total	2000 mg	2000 mg

### Analysis of efficacy on cognitive functions in subjects

**Figure 3:**
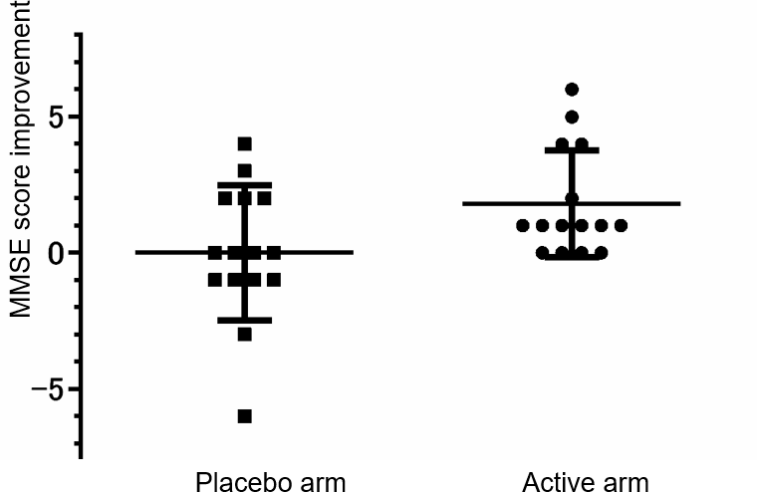
**The distribution of the MMSE scores change.** A dot shows the points of individual improvement. A bar shows the average in the placebo-administered subjects or the anserine-administered subjects and ± Standard Deviation.

Table 3 shows the results of the MMSE. We detected a significant difference between the active arm and the placebo arm in the primary outcome, the change value of MMSE scores (Two-way repeated ANOVA (Time x Treatment), F_(1,28)_ = 4.8462, p = 0.036). As shown in Figure 3, no subject in the active arm deteriorated the score of MMSE. In the placebo arm, two subjects fell below the cutoff score for dementia (23/24) of MMSE, and both of them reported symptoms that did not contradict the onset of dementia. We did not detect any difference in the change score of ADAScog between the two arms (p = 0.92). The score change of the active arm was -0.4 ± 3.2, and that of the placebo arm was -0.3 ± 2.6. To evaluate the contribution of daily intake of anserine or carnosine to the score change of MMSE, we performed a multiple regression analysis (Table 4; regression variation, p = 0.0014). A negative effect of the daily anserine intake (partial regression coefficient = -0.0109, p = 0.0209) and a positive effect of the daily carnosine intake were found on the improvement of the MMSE scores in the twelve weeks of anserine supplementation. The greater benefit of anserine supplementation was suggested in individuals taking less anserine from daily meals.

**Table 3:** MMSE psychometric test data for the participants who completed the trial.

	**Baseline**		**Follow-up**		**Change (Time x Treatment)**		
	**Active**	**Placebo**	**Active**	**Placebo**	**Active**	**Placebo**	**p value**
MMSE	25.5±2.1	26.9±2.8	27.3±1.8	26.9±3.0	1.8±2.0	0±2.5	0.036*

^a^*p* value less than 0.05 is shown by *.

**Table 4:** Multiple regression analysis of the change score of MMSE with the amount of anserine and carnosine.

**Variable**	**Partial regression coefficient**	**Standard error**	**Standard partial regression coefficient**	**F value**	**t value**	**p value**^a^
Active/Placebo (1/0)	1.5710	0.6214	0.4426	6.3915	2.5281	0.0176*
Anserine (mg/day)	-0.0109	0.0045	-1.8426	6.0140	-2.4524	0.0209*
Carnosine (mg/day)	0.0240	0.0087	2.0279	7.5088	2.7402	0.0107*

^a^p value less than 0.05 is shown by*.

### Clinical safety

A participant in the active arm had felt like heatstroke before the tests at baseline and rescheduled the participation, then scarcely ingested the test food and dropped out due to dizziness in the way. Four participants, comprised of two in the active arm and two in the placebo arm, dropped out due to unknown reasons during the intervention. No cause-and-effect relationship about adverse effects was reported or suggested.

### Effect of anserine treatment on the blood CRP-level

To investigate the mechanism of the anserine’s action in the brain, we tested fourteen elderly volunteers from the participants of the above-mentioned RCT for blood before and after the seven days of oral anserine supplementation. We noted a significant decrease in the blood CRP (p = 0.036, after the paired t-test). We did not observe significant differences in the level of MPO and the tau-P 181 after the anserine treatment (data not shown).

**Figure 4:**
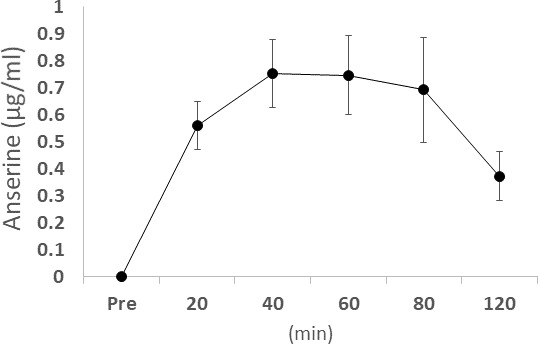
**The concentration of blood anserine after ingestion.** Dots and bars show the average ± Standard Deviation.

**Figure 5:**
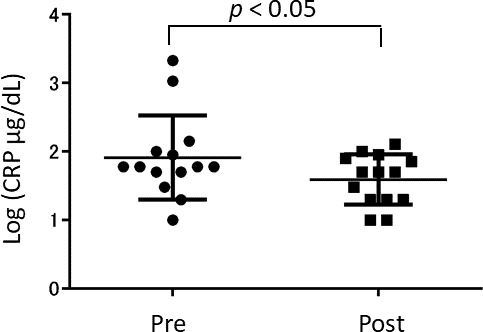
**The concentration of blood CRP (C-Reactive Protein) after the anserine treatment.** Dots show the data of the individuals. Bars show the average ± Standard Deviation.

In an independent study, elderly volunteers ingested 750 mg of anserine and 250 mg of carnosine and provided us venous blood samples. The plasma samples obtained before, 20 min, 40 min, 60 min, 80 min, and 120 min after the ingestion were tested for the anserine and carnosine levels. The maximum anserine concentration was reached between 40 and 60 minutes, and the half-life of anserine's blood concentration was shown to be about an hour (Figure 4), while the transfer of carnosine from the digestive system to the blood was not detected. We investigated the relationship between anserine's blood concentration and the change value of the serum CRP in the subjects of the anserine administration for a week (Figure 5). A significant correlation was detected between the blood anserine level and the decrease in CRP level (ΔCRP = -0.004 x (plasma anserine concentration) – 0.0665; n = 14, R2 = 0.79, p < 0.05).

## DISCUSSION

In the present study, we have shown for the first time that the effect of anserine, a natural HClO-scavenger, on the cognitive function in persons with MCI. Previous studies from our laboratory and others showed that anserine/carnosine supplementation at a dose of 1000 mg per day (anserine 750mg and carnosine 250mg) helps preserve cognitive function and keeps brain blood flow [29–34]. In our last trial [32], MMSE was used to evaluate the effect of anserine supplementation at a dose of 750 mg per day for 12 weeks. In the present trial, a lower dose of the possible active compound, anserine, was challenged. As a result, we detected a significant improvement with anserine (500 mg/day), as the change in the MMSE scores was 1.8 ± 2.0 in the active arm, 0 ± 2.5 in the placebo arm. Besides, daily anserine intake was found to affect the benefit of anserine supplementation. Very recently, in the Hisayama cohort, Hata et al. have demonstrated an inverse correlation between the risk of dementia onset and the serum level of beta-alanine, a metabolite of anserine [37]. In line with these observations, Toh et al. have revealed that anserine's daily consumption has favorable cognitive effects, especially in the memory domain, in a meta-analysis [38]. Therefore, it can be reasonably postulated that oral anserine supplementation benefits elderly individuals with the risk of dementia.

Neuroinflammation in the pathogenesis of AD has been attracting attention [8–10]. We have demonstrated that imidazoledipeptides control microglia appearing near the damaged brain tissues and take part in regulating inflammatory responses in a transgenic AD mouse model [36, 39]. However, the mechanism of controlling microglia had been unresolved. In a transgenic AD mouse model, it was well shown that the innate immune system, including neutrophils, contributes to the neuroinflammation and the memory decline in the AD mice [40, 41]. Zenaro et al. have reported that neutrophils adhered to vascular walls and enter brain tissue toward the senile plaques in the brain of AD model mice and AD patients. They found the accumulation of MPO around neutrophil extracellular traps (NETs) in the brain of AD model mice and AD patients. Very interestingly, they also demonstrated that the administration of antibodies against LFA-1, a molecule involved in the adsorption of neutrophils to the vascular walls, reduced the accumulation of senile plaques in brain tissue, subsided the activation of microglia, and improved memory function in the AD model mice [40]. Recently, Cruz Hernández et al. have reported transient clogging of micro-capillaries observed in AD model mouse brain through two-photon live imaging. Neutrophils, in collaboration with erythrocytes and platelets, were involved in this clogging. The administration of antibodies for one of the neutrophils' surface markers, Ly6g, improved the clogging and memory function shortly [41].

The facts of cognitive improvement by suppressing MPO activity in AD model mice considered together [19, 20], reasonable speculation could be that in the progression of AD, neutrophils adhere to neurovascular walls to release MPO, and MPO produces HClO radicals to generate neuroinflammation. In our previous studies, carnosine supplementation recovered the blood-vessel abnormality in these mice [39]. Anserine supplementation in AD model mice significantly prevented damage in brain microvascular pericytes [36]. Anserine has specific antioxidative activity against HClO radicals [42], as shown in Figure 2. In our previous study utilizing an MRI method, Arterial Spin Labeling (ASL), the ingestion of imidazole dipeptides for 3-12 months improved cerebral blood flow in the healthy elderly subjects [29, 30]. It has also been suggested that erythrocytes [43–45] or platelets [46] take in anserine, though the anserine level in the plasma just transiently increases after every oral intake [47, 48]. In our preliminary test of 1000 mg anserine supplementation per day with four adult healthy volunteers, the elevation of anserine concentration in platelets was observed by a high-spec LCmsms method, 45 min after the ingestion (CL, HL, TH; unpublished observation). Besides, several reports are suggesting that neutrophils take in imidazole dipeptides, anserine and carnosine, through PEPT2 [49], which transports the ligand of Nucleotide-binding Oligomerization Domain (NOD) receptor such as MurNAc-L-Ala-D-isoGlu (MDP) [50–52]. There are also a series of reports showing that imidazole dipeptides repel the toxicity of HClO generated by MPO [21, 42, 45, 53]. Therefore, we could reasonably speculate as follows; neutrophils, erythrocytes, or platelets take in anserine, then the anserine in these blood cells scavenges the HClO radicals produced by MPO from neutrophils in the brain capillaries, thereby suppressing blood flow stagnation to protect cognitive functions.

In line with our findings on anserine for AD, Peters et al. have very recently demonstrated that anserine ameliorated diabetic nephropathy and halved proteinuria in diabetic mice (db/db) [54]. They also reported that anserine has more vigorous antioxidative activity than carnosine [54, 55], which was consistent with the treatments for diabetic nephropathy with carnosine or its derivative [56–58]. In addition to AD and diabetes, imidazole dipeptides exert antioxidative effects against inflammation in various diseases such as renal nephropathy, retinal degeneration, or pneumonia [21, 59]. Their pathogenesis involves neutrophil-mediated MPO activities [60, 61]. Our pilot monitoring for the present trial revealed statistically significant improvement in the blood creatinine level among the ten healthy adult volunteers administered 500 mg of the anserine supplementation every day for 12 weeks (data not shown). It suggested a potential effect of anserine on renal microvasculature. Also, virus-induced acute lung injury and MPO-mediated septic shock were relieved with imidazole dipeptides in rodent models [62–64]. Evidence of anserine against acute or chronic inflammation may support the notion that oral anserine intake is useful for protecting against the harm of endogenous HClO.

In this communication, we have proposed that daily intake of anserine supplementation at a dose of 500 mg per day helps preserve cognitive functions in elderly individuals with MCI by suppressing HClO radicals' actions. There may be some complementary beneficial effects by the additives adopted, vitamin c or ferulic acids, to reinforce the inhibition of MPO-HClO-mediated inflammation by anserine. There are reports that both vitamin c and ferulic acid provide favorable effects for AD patients [65–68]. Their antioxidative activity against HClO may synergize with anserine since vitamin c removes ONOO- radicals and ferulic acids do HO- radicals (Figure 2).

Accordingly, in the brain of elderly individuals who may be apt to aggravate cognitive declines due to AD, the production of HClO radicals from NETs in the brain parenchyma surrounding senile plaques presumably damages not only astrocytes but also microglia to promote neuroinflammation. Before the penetration of neutrophils into the affected brain parenchyma, anserine in the intracellular spaces of neutrophils, platelets, and erythrocytes in the brain micro-capillaries may remove the harmful effect of HClO [69–72]. This study proposes the molecular strategy to control MPO-HClO-NETs cascade, leading to brain micro-capillaries' damage and the cognitive declines associated with AD dementia, by a natural antioxidant, anserine, that is a potent scavenger for HClO radicals.

### Limitations

In this study, the subject individuals were selected utilizing a cutoff value of the MoCA test [73], which is often used as a reference to diagnose MCI. However, we did not thoroughly examine the subtypes of MCI. There is a lack of experimental data regarding the incorporation of anserine into neutrophils after oral intake. In the present study, the efficacy of anserine was examined in three months. We would like to know how long this beneficial effect on cognitive functions will last. Long-term observation is awaited.

## CONCLUDING REMARKS

In this communication, we noted that anserine has a cognitive improvement effect in persons with MCI, probably through a mechanism in which anserine scavenges HClO radicals generated by MPO released from neutrophils in the brain microcapillaries.

## MATERIALS AND METHODS

### Study design

This study is a randomized, double-blind, placebo-controlled trial conducted to evaluate anserine's effects on community-dwelling individuals with MCI and in generally healthy physical condition. This study was approved by the Ethics Committee of The University of Tokyo (ID 17-218). This study was registered in the UMIN Clinical Trials Registry (UMIN000032319). The protocol of this study was in accordance with the Declaration of Helsinki and the Ethical Guidelines for Medical and Health Research Involving Human subjects. The participants were enrolled in the present trial by a responsible doctor. The randomizing allocation was planned to include equal numbers of subjects in the active arm and the placebo arm. To detect 1.5 point difference for the MMSE score with a type1 error protection of 0.05 and 80 % of power assuming a standard deviation (SD) of 1.5 from the results of the previous studies [32, 74], we performed a calculation to get a result that the number of necessary subjects in the present trial was 30. All of the study subjects and the clinical staff were blinded about the allocation through the follow-up test. The assignment to the two arms was performed by a third party (Imepro Inc., Tokyo, Japan), who also delivered the test formula to the participants.

### Participants

The present trial included 12 weeks for intervention that started in July 2018. The study participants were expected to be generally healthy and not demented. They walked to the cognitive testing site in the suburb of the Tokyo Metro Area. We invited some of the present trial participants because they had scored 25 or less on the Montreal cognitive assessment (MoCA) [73], more than a month previously. All the participants received a detailed explanation of the present study's purpose and procedure and provided written informed consent. The inclusion criteria were as follows: (1) the MoCA score is 25 or less at baseline [73], (2) a responsible doctor contradicted the diagnosis of dementia. The exclusion criteria were as follows: (1) acute or sub-acute illness of local brain lesion due to head injury, brain ischemia, or brain tumor, (2) the usage of donepezil, galantamine, rivastigmine, memantine, or imidazole dipeptides in the previous six months, (3) a history of a severe psychiatric illness or any obvious symptom or sign of psychiatric disorder at present, (4) the use of psychopharmaceuticals at present, other than sleep medication in the night, (5) allergy to salmon, (6) inability to walk to the test site, (7) participation or a plan to participate in another trial, (8) judgment of inadequacy to enroll by a responsible doctor [32].

### Inventory of anserine and carnosine in the everyday diet

A dietary survey was conducted using a semi-quantitative method reported elsewhere to estimate anserine and carnosine intake from the usual diet, as described previously [32]. At the baseline and follow-up, participants filled out a self-administered questionnaire on the frequency of animal meat (chicken, pork, and beef) or fish (divided into salmon, red-meat fish represented by tuna, white fish, blue-back fish represented by mackerel, and eel) in their diet during the previous 12 weeks.

### Test formulae

We started by considering the effects of imidazoledipeptides ingestion on their blood concentration. In an independent assay, elderly volunteers ingested 750 mg of anserine and 250 mg of carnosine. We sampled the plasma of venous blood before and after the ingestion. The plasma was deproteinized with trichloroacetic acid (final concentration 5%) and filtered through a 0.45 μm filter to prepare HPLC samples. The method by Dunnett and Harris was partially modified and applied to quantify histidine-related compounds [75]. Carnosine was hardly absorbed into the vascular system or rapidly disappeared from the blood after ingestion comparatively to anserine, described in Figure 4. Therefore, we tried to prepare a test food that includes anserine only as an imidazoledipeptide for planning the present trial. Salmon was chosen as the raw material as it is free of carnosine, but abundant in anserine [21]. Purification of the processed material through a cation-exchanger and nano-filtration unit, utilized in the previous study [32], enabled us to remove creatinine, as well as odors specific to fish products, from anserine powder. For evaluating antioxidative activities toward radicals, HClO radicals were prepared by diluting sodium hypochlorite solution with Dulbecco’s buffered saline pH 7.2 (Wako). OH radicals were prepared just before the protein degradation assay by modification of the Fenton reaction. Briefly, 1ml of 130mM H_2_O_2_ was added to 100 μl each of 100mM EDTA, 100mM FeCl_3_ Â· 2H_2_O. ONOO radicals were prepared by the quench reactor method, as described before [42]. For protein degradation assay to evaluate the antioxidative activities of each ingredient, egg white protein (Ovalbumin, Sigma Chemicals, St. Louis, MO) dissolved in buffered saline at a concentration of 2.5 mg/ml was used as the target protein. A 200-μl aliquot of protein solution was placed in a 1-ml centrifugal tube and mixed with 25μl of the solution containing each antioxidant. The final concentrations of the active ingredients were 5mM for anserine (Ans) and vitamin C (VC), and 0.5mM for ferulic acid (FA).

### Cognitive tests

To evaluate the cognitive function of participants, we utilized MMSE as a primary neuropsychological test. We obtained MMSE scores from every subject before and after the intervention period. We also obtained ADAS scores [30] to evaluate cognitive declines' progression related to dementia to apply the exclusion criteria.

### Safety evaluation

In the previous randomized controlled trial conducted for 12 weeks with MCI individuals [32], we observed no adverse effect considered to have a cause-and-effect relationship with anserine administration of more than 500 mg per day. Besides, we have preliminarily monitored ten healthy adult volunteers who ingested the active supplement containing 500 mg of anserine, the same amount as in the present trial, every day for 12 weeks. There was no adverse effect or significant exacerbation in the items of blood biochemistry tests. In the present study, a responsible doctor interviewed the individuals who came to the follow-up test concerning any symptoms they could have.

### Statistical analysis

To examine the effects of anserine supplementation on cognitive function, we performed a two-way repeated ANOVA (Time [baseline or follow-up] x Treatment [the active (anserine) or placebo]. A *p* value of less than 0.05 was defined as statistically significant. Data are shown as mean ± standard deviation (SD).

### Ethics approval and consent to participate

The study was approved by the Local Medical Ethics Committees (The University of Tokyo, Tokyo, Japan).

### Human and animal rights

All human procedures followed the Helsinki Declaration of 1975. No animals were used for this study.

### Author disclosures

Dr. Yanai, Mr. Shiotani, and Mr. Sato are employees of Tokai Bussan Co. Ms. Inamura is an employee of Mitsui Fudosan Co. Dr. Hisatsune reports grant support from Tokai Bussan Co. during the study. Authors not named here have disclosed no conflicts of interest.

### Consent for publication

All patients provided written informed consent for their data to be used for research purposes.
